# Efficient Parameter Estimation for Sparse SAR Imaging Based on Complex Image and Azimuth-Range Decouple

**DOI:** 10.3390/s19204549

**Published:** 2019-10-19

**Authors:** Mingqian Liu, Bingchen Zhang, Zhongqiu Xu, Yirong Wu

**Affiliations:** 1 Aerospace Information Research Institute, Chinese Academy of Sciences, Beijing 100094, China; zhangbc@aircas.ac.cn (B.Z.); xuzhongqiu18@mails.ucas.ac.cn (Z.X.); wyr@mail.ie.ac.cn (Y.W.); 2 School of Electronic, Electrical and Communication Engineering, University of Chinese Academy of Sciences, Beijing 101408, China; 3 Key Laboratory of Technology in Geo-Spatial Information Processing and Application System, Institute of Electronics, Chinese Academy of Sciences, Beijing 100190, China

**Keywords:** sparse synthetic aperture radar (SAR) imaging, adaptive parameter estimation, compressive sensing (CS), L_1_ regularization, azimuth-range decouple, Gaofen-3 data

## Abstract

Sparse signal processing theory has been applied to synthetic aperture radar (SAR) imaging. In compressive sensing (CS), the sparsity is usually considered as a known parameter. However, it is unknown practically. For many functions of CS, we need to know this parameter. Therefore, the estimation of sparsity is crucial for sparse SAR imaging. The sparsity is determined by the size of regularization parameter. Several methods have been presented for automatically estimating the regularization parameter, and have been applied to sparse SAR imaging. However, these methods are deduced based on an observation matrix, which will entail huge computational and memory costs. In this paper, to enhance the computational efficiency, an efficient adaptive parameter estimation method for sparse SAR imaging is proposed. The complex image-based sparse SAR imaging method only considers the threshold operation of the complex image, which can reduce the computational costs significantly. By utilizing this feature, the parameter is pre-estimated based on a complex image. In order to estimate the sparsity accurately, adaptive parameter estimation is then processed in the raw data domain, combining with the pre-estimated parameter and azimuth-range decouple operators. The proposed method can reduce the computational complexity from a quadratic square order to a linear logarithm order, which can be used in the large-scale scene. Simulated and Gaofen-3 SAR data processing results demonstrate the validity of the proposed method.

## 1. Introduction

Synthetic aperture radar (SAR) is an important imaging technology that has been applied in environmental protection and marine observation [1,2]. In recent years, the sparse signal processing method based on CS [3] has been implemented in microwave imaging [4,5]. It can recover the scene by solving $L_{q}(0<q\leq 1)$ regularization.

In [6], Çetin et al. proposed a sparsity-driven SAR imaging model for achieving autofocusing and moving targets imaging. Zhang et al. [7] explored the principles and applications in sparse microwave imaging. Patel et al. [8] analyzed different azimuth sampling methods based on the CS model. Luo et al. [9] developed a multiple scatterers detection method for SAR tomography with CS approach. Hossein et al. [10] proposed a polarimetric SAR estimator under the frame of CS. In [11], Zhu reviewed the CS-based super-resolving algorithm. Zhang et al. [12] proposed a novel 3D SAR imaging algorithm based on 2D compressive sensing. It not only provides super-resolution performance, but also reduces the storage of data acquisition and processing. Compared with matched filtering (MF), microwave imaging based on sparse signal processing can improve the image quality by suppressing noise and sidelobes as well as azimuth ambiguities with the downsampled data [7,13].

In compressive sensing (CS), the sparsity is usually considered as a given parameter. However, it is unknown practically. For many functions of CS, we need to know this parameter. Therefore, the estimation of sparsity is crucial for sparse SAR imaging. In some cases, the sparsity can be estimated directly based on prior information, which is obtained from the historical data. In other cases, we can only get the range of the sparsity based on the prior information, rather than an accurate value. In the process of accurately reconstructing a large number of these scenarios, it is more advantageous to estimate the sparsity automatically than to select the sparsity manually. In [14], several methods, such as Stein’s unbiased risk estimator, L-curve, and generalized cross-validation, have been presented for automatically estimating the regularization parameter. Adaptive parameter estimation for sparse SAR imaging can be achieved by these methods. However, these methods are deduced based on an observation matrix. The observation matrix-based sparse SAR imaging achieves decouplingby vectorizing the raw data matrix, which will entail huge computational and memory costs. Therefore, it is challenging to adopt these adaptive parameter estimation methods based on an observation matrix into a large-scale scene reconstruction.

An azimuth-range decouple-based sparse SAR imaging method has been proposed [7,15]. The coupling of the 2D data can be removed by constructing an echo simulation operator to replace the observation matrix, which can effectively relieve the computational complexity [16]. This method has been widely used in TOPS SAR [17], Sliding Spotlight SAR [18], displaced phase center antenna (DPCA) imaging [19], wide-angle SAR (WASAR) [20] and ground moving target indication (GMTI) [21]. We can combine it with automatic parameter estimating methods to achieve an adaptive parameter estimation of the large-scale sparse SAR imaging. However, considering that finding the optimal regularization parameter requires iterative processing, the total computational cost of the adaptive parameter estimation method based on azimuth-range decouple is still large.

A complex image-based sparse SAR imaging method is proposed in [22,23]. Combining this method with the automatic parameter estimating methods, we get the adaptive parameter estimation method based on a complex image. The complex image-based sparse SAR imaging method only considers the threshold operation of the complex image, which can further reduce the computational and memory costs. In this paper, for the case of the downsampled raw data, we propose an efficient adaptive parameter estimation method. The complex image-based sparse SAR imaging method is adopted first to pre-estimate the parameter. Then, the parameter iteration range is updated according to the pre-estimated parameter. Finally, we introduce the azimuth-range decouple operators into the parameter estimation and deduct the efficient adaptive parameter estimation method for sparse SAR imaging.

The rest of this paper is organized as follows. In Section 2, we introduce the sparse SAR signal models and the automatic regularization parameter estimation method. In Section 3, we give details of the proposed method. The computational complexity of different methods is also analyzed in this section. Section 4 presents the simulated and real data results to analyze the performance of the proposed method. The conclusions are presented in Section 5.

## 2. Signal Model and Automatic Parameter Estimation Method

### 2.1. Signal Model

The observation-matrix-based sparse SAR data formation model is represented as follows [7]:

(1) y=Φx+n,

where $y\in {\mathbb{C}}^{N\times 1}$ is the SAR echo data vector, N = $N_{a}$ (azimuth) × $N_{r}$ (range), $x\in {\mathbb{C}}^{N\times 1}$ is the backscattered coefficient vector, Φ is the observation matrix, and $n\in {\mathbb{C}}^{N\times 1}$ is the noise vector.

For the data formation model Equation (1), if the considered scene x is sparse enough and the observation matrix Φ satisfies the restricted isometry property (RIP) [24], x can be reconstructed by solving the $L_{1}$ optimization problem:

(2) $$\hat{x}=\underset{x}{\arg\min}\{{\left\|y-\Phi x\right\|}_{2}^{2}+\lambda {\left\|x\right\|}_{1}\},$$

where λ is the regularization parameter. There are many algorithms to solve (e.g., Equation (2)), such as the convex optimization algorithm [25], Bayesian learning algorithm [26], nonconvex optimization algorithm [27,28], and greedy algorithm [29]. After reconstruction, $\hat{x}$ is transferred to a matrix ${\hat{X}}_{\Phi }$.

#### 2.1.1. Azimuth-Range Decouple-Based Sparse SAR Imaging

The azimuth-range decouple-based sparse SAR imaging method is proposed in [7,15], the echo simulation operator G(·) is used to replace the observation matrix Φ, which is the inverse of the imaging operator I(·), that is, $G(\cdot )=I^{-1}(\cdot )\approx \Phi$. Then the azimuth-range decouple-based sparse SAR data formation model is represented as follows:

(3) $$Y=E_{a}G(X)E_{r}+N.$$

$E_{a}\in {\mathbb{C}}^{N_{a}\times N_{a}}$ is an azimuth downsampling matrix, $E_{r}\in {\mathbb{C}}^{N_{r}\times N_{r}}$ is a range downsampling matrix. Both $E_{a}$ and $E_{r}$ are the binary matrices to denote the downsampling strategy, which are no longer identity matrices, thus reducing the number of measurements. $Y\in {\mathbb{C}}^{N_{a}\times N_{r}}$ is the SAR raw data matrix, $X\in {\mathbb{C}}^{N_{a}\times N_{r}}$ is the backscattered coefficient matrix, and $N\in {\mathbb{C}}^{N_{a}\times N_{r}}$ is the noise matrix.

For this data formation model, the considered scene can be reconstructed by solving the $L_{1}$ optimization problem:

(4) $${\hat{X}}_{\lambda 1}=\underset{X}{\arg\min}\{{\left\|Y-E_{a}G(X)E_{r}\right\|}_{2}^{2}+\lambda {\left\|X\right\|}_{1}\},$$

where ${\left\|\;\cdot \;\right\|}_{2}$ is the 2-norm of a matrix.

#### 2.1.2. Complex Image-Based Sparse SAR Imaging

A complex image-based sparse SAR imaging method is proposed in [22]. This method first establishes the imaging model with the complex image after MF recovery as the input, then represents the reconstruction of sparse scene as an $L_{1}$ optimization problem, and finally utilizes the iterative recovery algorithm to get the focused high-resolution SAR imagery. The signal model is represented as follows:

(5) $$X_{MF}=X+N_{0},$$

where $X_{MF}\in {\mathbb{C}}^{N_{a}\times N_{r}}$ is the MF-reconstructed SAR complex image and $N_{0}\in {\mathbb{C}}^{N_{a}\times N_{r}}$ is the noise matrix.

For this model, the considered scene can also be reconstructed by solving the $L_{1}$ optimization problem:

(6) $${\hat{X}}_{\lambda 2}=\;\underset{X}{\arg\min}\{{\left\|X_{MF}-X\right\|}_{2}^{2}+\lambda {\left\|X\right\|}_{1}\}.$$

### 2.2. Automatic Parameter Estimation Method

Several methods have been presented for automatically estimating the regularization parameter. We choose the generalized cross-validation (GCV) method [14,30] as the parameter estimation method, which can estimate λ by minimizing the following cost function without knowing the noise variance:

(7) $$V_{\lambda }=\frac{\frac{1}{N}{\left\|\Phi {\hat{X}}_{\Phi }-Y\right\|}_{2}^{2}}{{\left[\frac{1}{N}tr(I-H_{\lambda })\right]}^{2}},$$

where $Y\in {\mathbb{C}}^{N_{a}\times N_{r}}$ is the SAR raw data matrix, N is the scene size, and tr(·) is the trace operator of a matrix. $H_{\lambda }$ is given in Equation (8):

(8) $$H_{\lambda }=\Phi {\left(2\Phi^{H}\Phi +\lambda M({\hat{X}}_{\lambda },\beta )\right)}^{-1}2\Phi^{H}.$$

In (8), $M({\hat{X}}_{\lambda },\beta )$ is a diagonal matrix whose ith diagonal element is $\beta {\left({\left|{\left({\hat{x}}_{\lambda }\right)}_{i}\right|}^{2}+\beta \right)}^{-3/2}$, where ${\hat{x}}_{\lambda }=\mathrm{vec}({\hat{X}}_{\Phi })\in {\mathbb{C}}^{N\times 1}$ and β is a small positive constant.

## 3. Efficient Adaptive Parameter Estimation for Sparse SAR Imaging

In this section, the parameter estimation method based on azimuth-range decouple and the parameter estimation method based on complex image are introduced. Next, we introduce the proposed method in detail. Finally, the computational complexity of these methods is analyzed.

### 3.1. The Adaptive Parameter Estimation Method Based on Azimuth-Range Decouple

Combining the azimuth-range decouple operators with GCV, we can get the adaptive parameter estimation method for sparse SAR imaging. Compared with the adaptive parameter estimation method based on observation matrix, this method can reduce the computational complexity. Considering that $M({\hat{X}}_{\lambda },\beta )$ is a large diagonal matrix of N×N, the computational cost of the trace of it is also large, we replace the trace operator tr(·) with the sum operator. Equation (7) can be rewritten as follows:

(9) $$V_{\lambda 1}=\frac{\frac{1}{N}{\left\|G({\hat{X}}_{\lambda 1})-Y\right\|}_{2}^{2}}{{\left[\frac{1}{N}\sum_{i=1}^{N_{a}}\sum_{j=1}^{N_{r}}\left(1-\frac{2}{2+\lambda \beta {\left({\left|{\left({\hat{X}}_{\lambda 1}\right)}_{ij}\right|}^{2}+\beta \right)}^{-3/2}}\right)\right]}^{2}},$$

which is the cost function of the adaptive parameter estimation method based on azimuth-range decouple, where G(·) is the echo simulation operator and ${\hat{X}}_{\lambda 1}$ is shown in Equation (4).

There are several algorithms to achieve the sparse reconstruction, such as iterative soft thresholding (IST) [31] and complex approximated message passing (CAMP) [32,33]. In this paper, we choose CAMP as sparse reconstruction algorithm, which has been applied to constant false-alarm rate (CFAR) detection in sparse SAR imaging [34].

The optimal regularization parameter is estimated by minimizing Equation (9). However, considering that finding the optimal regularization parameter requires the iterative processing, the total computational cost of the adaptive parameter estimation method based on azimuth-range decouple is still large.

### 3.2. The Adaptive Parameter Estimation Method Based on Complex Image

Compared with the azimuth-range decouple-based sparse SAR imaging method, the complex image-based sparse SAR imaging method only considers the threshold operation, which can further reduce the computational and memory costs. Combining it with GCV, we can get the adaptive parameter estimation method for sparse SAR imaging based on complex image. Equation (7) can be rewritten as follows:

(10) $$V_{\lambda 2}=\frac{\frac{1}{N}{\left\|{\hat{X}}_{\lambda 2}-X_{MF}\right\|}_{2}^{2}}{{\left[\frac{1}{N}\sum_{i=1}^{N_{a}}\sum_{j=1}^{N_{r}}\left(1-\frac{2}{2+\lambda \beta {\left({\left|{\left({\hat{X}}_{\lambda 2}\right)}_{ij}\right|}^{2}+\beta \right)}^{-3/2}}\right)\right]}^{2}},$$

which is the cost function of the adaptive parameter estimation method based on complex image, where ${\hat{X}}_{\lambda 2}$ is shown in Equation (6).

### 3.3. The Proposed Method

The proposed method is mainly for the case of the downsampled data. On the one hand, although the adaptive parameter estimation method based on azimuth-range decouple can estimate the sparsity accurately, as mentioned above, the total computational cost of this method is large. On the other hand, due to the energy dispersion and ambiguities, the estimated sparsity of the parameter estimation method based on complex image will be greater than the true value, and we cannot simply use the parameter estimation method based on complex image to replace the parameter estimation method based on azimuth-range decouple. Therefore, we need to find a method to adaptively estimate the sparsity accurately while having the lower computational complexity. A good solution is to combine these two adaptive methods together, utilizing the complex image to pre-estimate the parameter and reduce the iteration range, then estimating the accurate parameter with raw data.

The proposed method has three steps. First, set the iteration range of sparsity to $\left[K_{\min},K_{\max}\right]$ and adaptively estimate the sparsity based on the complex SAR image which is reconstructed by the downsampled raw data. The pre-estimated sparsity is set to $K_{\mathrm{mid}}$, which is greater than the true value due to ambiguities and energy dispersion caused by downsampling. Second, update the iteration range from $\left[K_{\min},K_{\max}\right]$ to $\left[K_{\min},K_{\mathrm{mid}}\right]$. Third, get the adaptive reconstructed image and the optimal adaptive result of sparsity $K_{opt}$ on the new range $\left[K_{\min},K_{\mathrm{mid}}\right]$ based on raw data.

**Figure 1:**
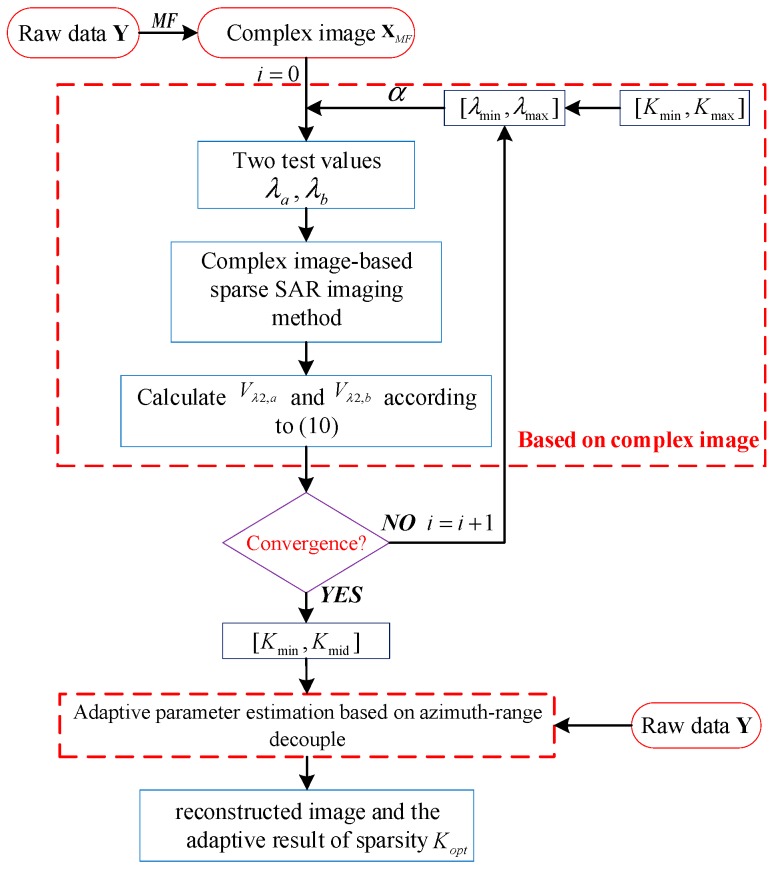
The flowchart of the proposed method.

The flowchart is shown in Figure 1.

The details of adaptive parameter estimation based on azimuth-range decouple are shown in Algorithm 1, where $\left[K_{\min},K_{\mathrm{mid}}\right]$ is the range of the sparsity; $\eta_{\lambda ,\mu ,CAMP}(\cdot )$ is the threshold function of CAMP.

**Algorithm 1:** The adaptive parameter estimation method based on azimuth-range decouple1: **Input:** downsampled SAR raw data Y, parameter δ,ε,μ, $\left[K_{\min},K_{\mathrm{mid}}\right]$
2:  **Initialization**: i=0, ${\hat{X}}_{\lambda 1}{}^{\left(0\right)}=0$, $\left[\lambda_{\min},\lambda_{\max}]\leftarrow [K_{\min},K_{\mathrm{mid}}\right]$
3:  **while**
$\log_{10}\lambda_{\max}-\log_{10}\lambda_{\min}>\varepsilon$
**and**
i<Iter
  1) $\lambda_{a}=10^{\log_{10}\lambda_{\min}+(1-\alpha )(\log_{10}\lambda_{\max}-\log_{10}\lambda_{\min})}$; $\lambda_{b}=10^{\log_{10}\lambda_{\min}+\alpha (\log_{10}\lambda_{\max}-\log_{10}\lambda_{\min})}$  2) ${\tilde{X}}_{\lambda 1,a}=Y-G\left({\hat{X}}_{\lambda 1,a}\right)+\frac{1}{2\delta }\cdot \left[\langle \frac{\partial \eta^{R}}{\partial x^{R}}({\tilde{X}}_{\lambda 1,a};\mu )\rangle +\langle \frac{\partial \eta^{I}}{\partial x^{I}}({\tilde{X}}_{\lambda 1,a};\mu )\rangle \right]+I({\hat{X}}_{\lambda 1,a})$;
  ${\tilde{X}}_{\lambda 1,b}=Y-G\left({\hat{X}}_{\lambda 1,b}\right)+\frac{1}{2\delta }\cdot \left[\langle \frac{\partial \eta^{R}}{\partial x^{R}}({\tilde{X}}_{\lambda 1,b};\mu )\rangle +\langle \frac{\partial \eta^{I}}{\partial x^{I}}({\tilde{X}}_{\lambda 1,b};\mu )\rangle \right]+I({\hat{X}}_{\lambda 1,b})$
  3) ${\hat{X}}_{\lambda 1,a}=\eta_{\lambda_{a},\mu ,CAMP}({\tilde{X}}_{\lambda 1,a})$; ${\hat{X}}_{\lambda 1,b}=\eta_{\lambda_{b},\mu ,CAMP}({\tilde{X}}_{\lambda 1,b})$
  4) Calculate $V_{\lambda 1,a}$ and $V_{\lambda 1,b}$ according to (9)  5) **if**
$V_{\lambda 1,b}>V_{\lambda 1,a}$
$\lambda_{\max}=\lambda_{b}$
**else**
$\lambda_{\min}=\lambda_{a}$
  6) i=i+14:  **end while**5: **Output**: the reconstructed image ${\hat{X}}_{\lambda 1,a}$ and the adaptive parameter $\lambda_{a}$


### 3.4. Analysis of Computational Complexity

The computational complexity of different adaptive parameter estimation methods is analyzed in this section. A common characteristic of the adaptive parameter estimation methods mentioned above is that regularization parameter iterations are required. The difference lies in the different sparse reconstruction algorithms.

The measure of the computational complexity is the floating point operation (FLOP). Each FLOP represents a real addition operation or a real multiplication operation. In the observation matrix-based sparse SAR imaging method and azimuth-range decouple-based sparse SAR imaging method, the main calculation includes the imaging process, the echo simulation process, and the threshold process. The computational complexity of the threshold process is $(8n+n\log_{2}n)$ FLOPs, where $n=N_{a}\times N_{r}$ is the scene size. In the observation matrix-based sparse SAR imaging method, the imaging process and echo simulation process are two matrix multiplications. The main computational complexity of a single-step iteration of the observation-matrix-based sparse SAR imaging method is $(16mn+8n+n\log_{2}n)$ FLOPs, where m is the sampling number. This computational complexity is approximately proportional to the quadratic square of the scene size.

In this paper, the chirp scaling [35] operator is chosen as the imaging operator. Therefore, I(·) and G(·) can be expressed as follows:

(11) $$I\left(Y\right)=F_{a}^{-1}\left(F_{a}Y⊙\Theta_{sc}F_{r}⊙\Theta_{rc}F_{r}^{-1}⊙\Theta_{ac}\right)$$

(12) $$G\left(X\right)=F_{a}^{-1}\left(F_{a}X⊙\Theta_{ac}^{*}F_{r}⊙\Theta_{rc}^{*}F_{r}^{-1}⊙\Theta_{sc}^{*}\;\right),$$

where $F_{a}$ and $F_{a}^{-1}$ are the azimuth Fourier transform (FFT) operators and azimuth inverse Fourier transform (IFFT) operators, $F_{r}$ and $F_{r}^{-1}$ are the range FFT operators and range IFFT operators, $\Theta_{sc}$, $\Theta_{rc}$ and $\Theta_{ac}$ are three complex phase matrix. Chirp scaling and inverse chirp scaling both contain two FFTs, two IFFTs, and three time complex phase multiplications. According to [2], the computational complexity of FFT and IFFT with length $l_{0}$ is $\left(5l_{0}\log_{2}l_{0}\right)$ FLOPs, and the computational complexity of a complex multiplication operation is six FLOPs. Assuming that the data are sampled in the manner of uniform/nonuniform downsampling, the main computational complexity of a single-step iteration of the azimuth-range decouple-based sparse SAR imaging method is $(46n+2m+21n\log_{2}n)$ FLOPs, which is approximately proportional to the product of the linear logarithm of the scene size.

The complex image-based sparse SAR imaging method includes only threshold process. The computational complexity of a single-step iteration of this method is $(8n+n\log_{2}n)$ FLOPs, which is much lower than the azimuth-range decouple-based sparse SAR imaging method.

Let I represent the iteration steps of the recovery for sparse reconstruction algorithms. Let J and $J_{2}$ denote the number of iteration steps required for regularization parameter convergence when the iteration ranges of sparsity are $\left[K_{\min},K_{\max}\right]$ and $\left[K_{\min},K_{\mathrm{mid}}\right]$, respectively. Assuming that I=20, J=16, $J_{2}=J/4$, the scene size n=4096×4096, and the downsampling rate m/n=80%, the computational complexity of different adaptive parameter estimation methods is shown in Table 1.

Since the proposed method utilizes the complex image as the prior information to pre-estimate the parameter, the iteration range of the sparsity is reduced when the adaptive parameter estimation is processed in the raw data domain. Therefore, the proposed method has the lower computational complexity compared with the parameter estimation method based on azimuth-range decouple. For example, if the scene size is 4096×4096 and the downsampling rate is 80%, the proposed method can increase the computational efficiency about 3‒4-fold.

## 4. Experiments

In this section, both simulation and real data experiments have been carried out to validate the effectiveness of the proposed method. The 1D simulation experiments compare the performance and reconstruction precision of the parameter estimation method based on observation matrix, parameter estimation method based on complex image and the proposed method. The 2D simulation experiments compare the adaptive result and computational complexity of different adaptive parameter estimation methods. Airborne SAR data and Gaofen-3 SAR data experiments are done to validate the ability of the proposed method to suppress energy dispersion and ambiguities. At last, the computational complexity of different adaptive parameter estimation methods is compared for different scene size.

### 4.1. 1D Simulation

**Figure 2:**
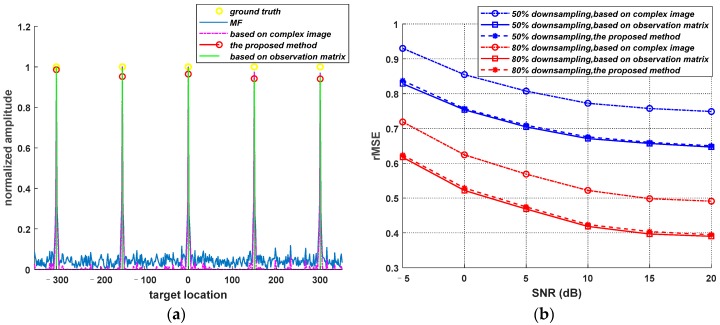
1D simulation results. (**a**) The reconstructed images obtained by MF, parameter estimation method based on observation matrix, parameter estimation method based on complex image and the proposed method. (**b**) The RMSE curves of three parameter estimation methods at different SNR and downsampling rate.

To validate the effectiveness of the proposed method, 1D simulation experiments are carried out. We set five point targets. Figure 2a shows the reconstructed images obtained by MF, parameter estimation method based on observation matrix, parameter estimation method based on complex image and the proposed method. In Figure 2a, the signal-to-noise ratio (SNR) is 15 dB and the downsampling rate is 80%. The adaptive $\lambda_{opt}$ of the adaptive parameter estimation method based on the observation matrix is 0.10 and the adaptive $\lambda_{opt}$ of the proposed method is 0.09. Due to downsampling, the adaptive λ of the adaptive parameter estimation method based on a complex image is 0.06, which is smaller than other two methods. From Figure 2a, we can conclude that the proposed method can effectively suppress the sidelobes and energy dispersion, and can recover the positions of target accurately compared with the positions of the ground truth. L_1_ regularization is known as a biased estimator [36,37], and the bias would underestimate the intensities of the targets. Therefore, in Figure 2a, the target amplitude of the proposed method is lower than the ground truth.

In order to explore the accuracy of different adaptive parameter estimation methods, Figure 2b shows the relative mean square error (RMSE) curves of three methods at different SNR and downsampling rate, where the downsampling rate are 50% and 80%, respectively. It can be seen from Figure 2b that the proposed method has the similar sparse recovery performance as the adaptive method based on an observation matrix. The reconstruction precision of the adaptive method based on a complex image is worse than other two methods when the raw data are downsampled.

### 4.2. 2D Simulation

**Figure 3:**
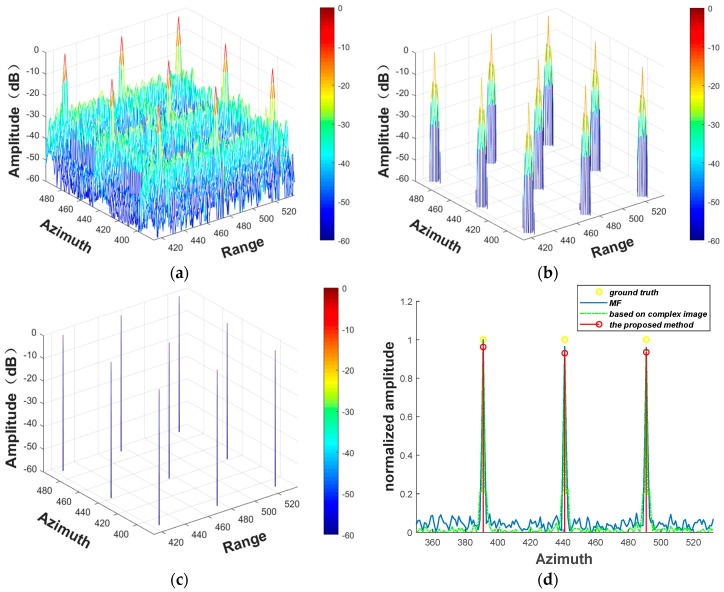
2D simulation results. (**a**) The reconstructed images obtained by MF. (**b**) The image reconstructed by parameter estimation method based on complex image. (**c**) The image reconstructed by the proposed method. (**d**) The azimuth profile.

In order to further analyze the effectiveness of the proposed method, 2D simulation experiments are carried out. The major simulation parameters are given in Table 2. The imaging results of nine point targets are shown in Figure 3. In Figure 3, the signal-to-noise ratio (SNR) is 20 dB and the downsampling rate is 80%. Figure 3a shows the image reconstructed by MF. Figure 3b shows the image reconstructed by adaptive parameter estimation method based on complex image, with the adaptive result λ being 0.17. From Figure 3b, we can see that the adaptive result based on complex image is not accurate when the raw data are downsampled, with the sidelobes still existing. Figure 3c shows the image reconstructed by the proposed method, with the adaptive result $\lambda_{opt}$ being 0.32. To better compare the reconstruction results of different methods, Figure 3d shows the azimuth profile of the 2D simulation experiment. Due to the bias of L_1_ regularization, in Figure 3d, the target amplitude of the proposed method is lower than the ground truth.

2D simulation experiments with different SNR and downsampling rates are also carried out. Table 3 shows the adaptive λ and RMSE of the parameter estimation method based on azimuth-range decouple, the parameter estimation method based on complex image, and the proposed method, respectively. According to Table 4, the adaptive λ of the adaptive parameter estimation method based on complex image is smaller than other two methods when the raw data are downsampled, and varies with the SNR and downsampling rate. We can also conclude that the adaptive parameter estimation method based on azimuth-range decouple and the proposed method have almost the same sparse recovery performance. With the decrease in the downsampling rate, the RMSE of different adaptive parameter estimation methods increases. Therefore, the downsampling rate is crucial for the reconstruction accuracy of the adaptive parameter estimation methods. In this experiment, when the downsampling rate is 80% and the SNR is 25 dB, the proposed method has the smallest RMSE, which is the best result.

**Figure 4:**
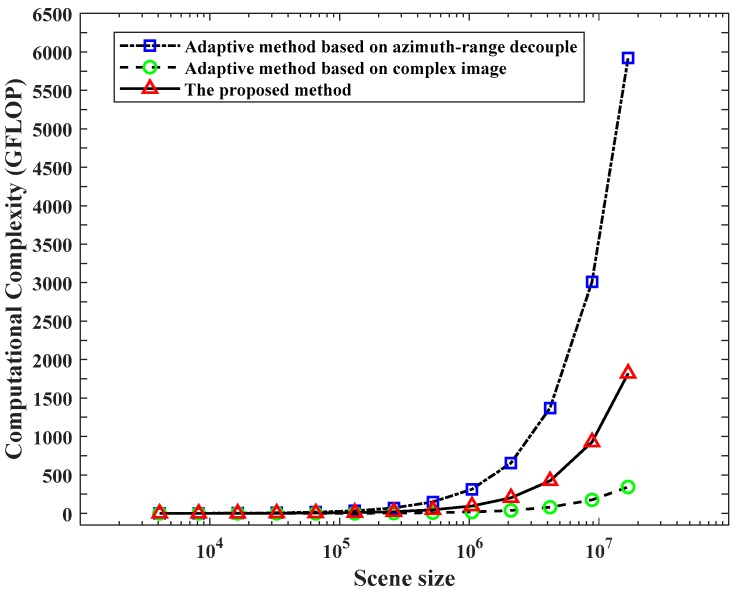
The computational complexity of different parameter estimation methods.

Next, we will analyze the computational complexity. To illustrate that the proposed method has lower computational complexity, the computational complexity of different adaptive parameter estimation methods is compared for different scene size is represented in Figure 4.

Figure 4 illustrates the computational complexity of three different adaptive parameter estimation methods for different scene size clearly. If the size of scene is over 1024×1024, the computational complexity of the adaptive parameter estimation method based on azimuth-range decouple increases dramatically. Although the computational complexity of the adaptive parameter estimation method based on complex image is the lowest, the adaptive result of this method is not accurate when the raw data are downsampled, as shown in Table 4. The proposed method utilizes complex image as prior information, thus having the lower computational complexity compared with the adaptive parameter estimation method based on azimuth-range decouple.

### 4.3. Airborne Data

**Figure 5:**
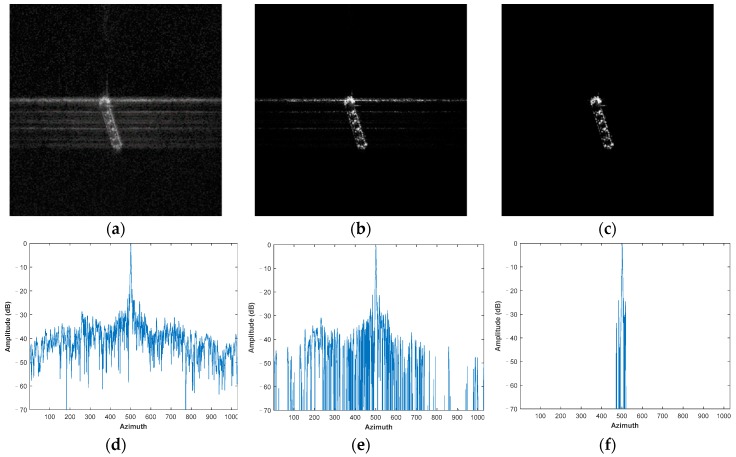
Airborne SAR data imaging results. (**a**) MF. (**b**) Parameter estimation method based on complex image, with $K_{mid}$ = 0.21. (**c**) The proposed method, with $K_{opt}$ = 0.02. (**d**) The azimuth profile of the imaging result of MF, with ISLR being −6.59 dB. (**e**) The azimuth profile of the imaging result of the adaptive parameter estimation method based on complex image, with ISLR being −9.14 dB. (**f**) The azimuth profile of the imaging result of the proposed method, with ISLR being −10.55 dB.

The airborne SAR data processing results are shown in Figure 5. The raw data are 80% randomly downsampled, received by the C-band airborne SAR system of Institute of Electronics, Chinese Academy of Sciences. The accurate sparsity of this scene is 0.02.

In order to better evaluate the performance of different adaptive methods, the integrated sidelobe ratio (ISLR) is chosen to quantitatively measure the ability to suppress the energy dispersion [2]:

(13) $${\mathrm{ISLR}=10\log}_{10}\left\{\frac{P_{\mathrm{total}}-P_{\mathrm{main}}}{P_{\mathrm{main}}}\right\},$$

where $P_{\mathrm{main}}$ is the main-lobe power, $P_{\mathrm{total}}$ is the total power.

Figure 5a shows the image reconstructed by MF, with the obvious energy dispersion, and Figure 5d is the azimuth profile of the imaging result of MF, with the ISLR being −6.59 dB. Figure 5b shows the imaging result of the adaptive parameter estimation method based on complex image, with the adaptive result of sparsity $K_{\mathrm{mid}}$ = 0.21. Figure 5e is the corresponding azimuth profile, with ISLR being −9.14 dB. From Figure 5b,e, we can see that when the raw data are downsampled, the adaptive parameter estimation method based on complex image cannot obtain an accurate result, with energy dispersion still existing. Figure 5c is the imaging result of the proposed method, with the adaptive result of sparsity $K_{opt}$ = 0.02, which converges to the accurate sparsity of the scene. Figure 5f is the azimuth profile of the imaging result of the proposed method, with ISLR being −10.55 dB. The proposed method can accurately estimate the sparsity and effectively suppress the noise and energy dispersion.

### 4.4. Gaofen-3 Data

**Figure 6:**
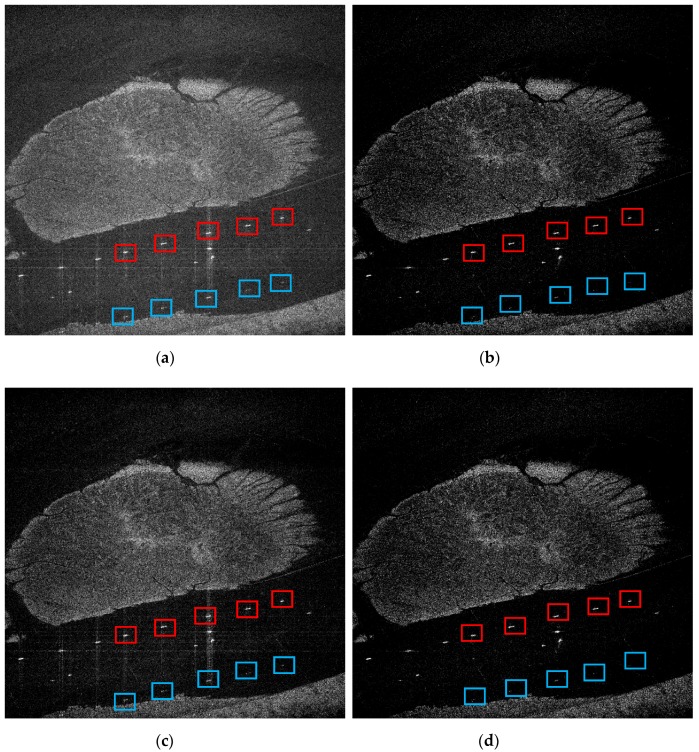
Gaofen-3 data imaging results. (**a**) MF. (**b**) Parameter estimation method based on azimuth-range decouple, with $K_{opt}$ = 0.3514. (**c**) Parameter estimation method based on complex image, with $K_{\mathrm{mid}}$ = 0.46. (**d**) The proposed method, with $K_{opt}$ = 0.3522.

The proposed method is also applicable to the spaceborne data. The Gaofen-3 satellite is a remote sensing satellite of China’s high-resolution special project, which was launched in August 2016. It is the first C-band multipolarized SAR imaging satellite with a resolution of 1 m. Gaofen-3 data are processed to verify the background clutter and noise suppressing ability and ambiguity suppressing ability of the proposed method. In this experiment, we perform 80% random downsampling for the fully sampled data. The Gaofen-3 data processing results are shown in Figure 6.

Figure 6a gives the MF imaging results of the downsampled raw data, with the obvious energy dispersion and azimuth ambiguities. Figure 6b shows the imaging result of the adaptive parameter estimation method based on azimuth-range decouple, with the adaptive result of sparsity $K_{opt}$ = 0.3514. It can be seen that this method can reconstruct the scene successfully and suppress the noise, energy dispersion and ambiguities efficiently. Figure 6c shows the imaging result of the adaptive parameter estimation method based on complex image, with the adaptive result of sparsity $K_{\mathrm{mid}}$ = 0.46. From Figure 6c, we can see that the adaptive result based on complex image is not accurate, with energy dispersion still existing. These two experimental results prove that the parameter estimation method based on azimuth-range decouple and the parameter estimation method based on complex image are not equivalent when the raw data are downsampled. However, we can use this pre-estimated sparsity as prior information to reduce the iteration ranges. Figure 6d is the imaging result of the proposed method, with the adaptive result of sparsity $K_{opt}$ = 0.3522, which is basically the same with the adaptive parameter estimation method based on azimuth-range decouple.

To further evaluate the ability of different adaptive methods to suppress the noise and ambiguity, target-to-background ratio (TBR) [38] and azimuth ambiguity-to-signal ratio (AASR) [23] are selected as two evaluation indicators. Their discrete expressions are defined as follows:

(14) $$\mathrm{TBR}(X{)=20\log}_{10}\left(\frac{\max_{(p,q)\in T}\left|X_{\left(p,q\right)}\right|}{1/N_{B}\sum_{(p,q)\in B}\left|X_{\left(p,q\right)}\right|}\right),$$

where B is the background area, $N_{B}$ is the pixel number in B, and T is the target region.

(15) $$\mathrm{AASR}=10\log_{10}\left(\frac{\frac{1}{N_{m}}\sum_{(p,q)\in M_{a}}{\left|X_{\left(p,q\right)}\right|}^{2}}{\frac{1}{N_{a}}\sum_{(p,q)\in A}{\left|X_{\left(p,q\right)}\right|}^{2}}\right),$$

where A is target region, $N_{a}$ is the pixels number in A, $M_{a}$ is the ambiguity area, and $N_{m}$ is the pixel number in $M_{a}$.

In this experiment, we chose five ships as performance test regions, as shown in the corresponding red frames. These five ships are represented as Ship 1–5, from left to right. Their corresponding azimuth ambiguity areas are shown in the blue frames.

The TBR of these five ships reconstructed by different methods are shown in Table 4. It can be seen from Table 5 that the proposed method can suppress the noise and energy dispersion effectively when the raw data are downsampled.

The AASR of these five ships reconstructed by different methods are shown in Table 5. From Table 5, we can see that the adaptive parameter estimation method based on complex image cannot suppress the azimuth ambiguity effectively. As a contrast, the adaptive parameter estimation method based on azimuth-range decouple and the proposed method both have the ability to decrease the azimuth ambiguity-to-signal ratio.

It can be seen from Table 4 and Table 5 that the adaptive parameter estimation method based on azimuth-range decouple and the proposed method have almost the same sparse recovery performance. According to the previous analysis and the simulation experiments, the proposed method has the lower computational complexity, which can be used in the large-scale scene.

## 5. Conclusions

In this paper, an efficient adaptive parameter estimation method for sparse SAR imaging based on complex image and azimuth-range decouple is proposed. The proposed method combines the advantages of the azimuth-range decouple-based sparse SAR imaging and the complex image-based sparse SAR imaging method. In the proposed method, the parameter is pre-estimated based on the complex image. Adaptive parameter estimation is then processed in the raw data domain combining with the pre-estimated parameter and azimuth-range decouple operators. Compared with the adaptive parameter estimation method based on complex image, the proposed method can estimate the sparsity accurately when the raw data are downsampled. Compared with the adaptive parameter estimation method based on azimuth-range decouple, the proposed method has the lower computational complexity, which can be used in the large-scale scene. The simulation, airborne SAR data and Gaofen-3 SAR data experiment results demonstrate its validity.

## Figures and Tables

**Table 1:** The computational complexity of different adaptive parameter estimation methods.

Adaptive Parameter Estimation Methods	Computational Complexity (FLOP)	Example (GFLOP)
Observation Matrix	$2JI\;(16mn+8n+n\log_{2}n)$	$2.31\times 10^{9}$
Azimuth-Range Decouple	$2JI\;(46n+2m+21n\log_{2}n)$	$5.92\times 10^{3}$
Complex Image	$2JI\;(8n+n\log_{2}n)$	$3.44\times 10^{2}$
The Proposed Method	$JI\;(39n+m+12.5n\log_{2}n)$	$1.82\times 10^{3}$

**Table 2:** Major parameters.

Parameters	Value
Center frequency	5.3 GHz
Pulse duration	2.5 μs
Velocity	70 m/s
Bandwidth	50 MHz
Sampling rate	60 MHz
Pulse repetition frequency (PRF)	130 Hz
Minimum slant range	3500 m

**Table 3:** The adaptive λ and RMSE for different SNR and downsampling rate.

Downsampling Rate	SNR (dB)	Adaptive λ and RMSE of Different Adaptive Parameter Estimation Methods					
		Azimuth-Range Decouple		Complex Image		The Proposed Method	
		λ	RMSE	λ	RMSE	λ	RMSE
80%	5	0.3178	0.6714	0.0734	0.8329	0.3190	0.6685
	10	0.3204	0.5932	0.0895	0.7762	0.3216	0.5833
	15	0.3216	0.5265	0.1282	0.6822	0.3221	0.5254
	20	0.3235	0.4887	0.1755	0.6345	0.3242	0.4855
	25	0.3237	0.4793	0.2130	0.6109	0.3245	0.4720
60%	5	0.2950	0.7944	0.0586	0.9364	0.3031	0.7883
	10	0.3082	0.7146	0.0842	0.8507	0.3127	0.7071
	15	0.3128	0.6231	0.1153	0.7926	0.3159	0.6205
	20	0.3194	0.5654	0.1483	0.7218	0.3206	0.5611
	25	0.3203	0.5590	0.1967	0.7023	0.3211	0.5528

**Table 4:** TBR of target regions based on different methods with downsampled data (80% downsampling).

Methods	Target-to-Background Ratio (dB)				
	Ship 1	Ship 2	Ship 3	Ship 4	Ship 5
MF	32.09	37.28	35.30	38.53	38.48
Based on azimuth-range decouple	47.61	52.26	49.72	54.08	55.71
Based on complex image	42.24	44.46	41.71	46.74	47.57
The proposed method	47.49	51.89	49.46	53.76	55.31

**Table 5:** AASR of target regions based on different methods with downsampled data (80% downsampling).

Methods	Azimuth Ambiguity-to-Signal Ratio (dB)				
	Ship 1	Ship 2	Ship 3	Ship 4	Ship 5
MF	−11.32	−12.02	−12.13	−11.30	−11.36
Based on azimuth-range decouple	−19.62	−20.10	−18.95	−21.66	−22.23
Based on complex image	−12.75	−13.92	−13.45	−12.70	−13.10
The proposed method	−19.43	−20.23	−18.62	−21.25	−22.24
